# Recent advances in epicutaneous immunotherapy and potential applications in food allergy

**DOI:** 10.3389/falgy.2023.1290003

**Published:** 2023-10-27

**Authors:** Pierre-Louis Hervé, Vincent Dioszeghy, Katie Matthews, Katharine J. Bee, Dianne E. Campbell, Hugh A. Sampson

**Affiliations:** ^1^ DBV Technologies, Montrouge, France; ^2^ DBV Technologies, Basking Ridge, NJ, United States; ^3^ Department of Allergy and Immunology, University of Sydney, Sydney, NSW, Australia; ^4^ Division of Allergy and Immunology, Icahn School of Medicine at Mount Sinai, New York, NY, United States

**Keywords:** epicutaneous immunotherapy, food allergy, desensitization, skin immunology, Langerhans cells, immune tolerance, Viaskin

## Abstract

Given the potent immunological properties of the skin, epicutaneous immunotherapy (EPIT) emerges as a promising treatment approach for inducing immune tolerance, particularly for food allergies. Targeting the highly immunocompetent, non-vascularized epidermis allows for the application of microgram amounts of allergen while significantly reducing the risk of allergen passage into the bloodstream, thus limiting systemic allergen exposure and distribution. This makes EPIT highly suitable for the treatment of potentially life-threatening allergies such as food allergies. Multiple approaches to EPIT are currently under investigation for the treatment of food allergy, and these include the use of allergen-coated microneedles, application of allergen on the skin pretreated by tape stripping, abrasion or laser-mediated microperforation, or the application of allergen on the intact skin using an occlusive epicutaneous system. To date, the most clinically advanced approach to EPIT is the Viaskin technology platform. Viaskin is an occlusive epicutaneous system (patch) containing dried native allergen extracts, without adjuvants, which relies on frequent application for the progressive passage of small amounts of allergen to the epidermis through occlusion of the intact skin. Numerous preclinical studies of Viaskin have demonstrated that this particular approach to EPIT can induce potent and long-lasting T-regulatory cells with broad homing capabilities, which can exert their suppressive effects in multiple organs and ameliorate immune responses from different routes of allergen exposure. Clinical trials of the Viaskin patch have studied the efficacy and safety for the treatment of life-threatening allergies in younger patients, at an age when allergic diseases start to occur. Moreover, this treatment approach is designed to provide a non-invasive therapy with no restrictions on daily activities. Taken together, the preclinical and clinical data on the use of EPIT support the continued investigation of this therapeutic approach to provide improved treatment options for patients with allergic disorders in the near future.

## Introduction

The investigation of immunotherapeutic strategies has increased exponentially since the rapid rise in the prevalence of food allergy at the turn of the century. Although the first report of food allergy immunotherapy for eggs dates back more than a century, only small case reports on the use of oral immunotherapy (OIT) appeared sporadically in the literature until 1998, when Patriarca et al. published the first in a series of reports demonstrating that a protocol of oral therapy could be effective in desensitizing children with immunoglobulin E (IgE)-mediated food allergy (1, 2). Numerous clinical trials proving the efficacy of OIT in desensitizing patients to a variety of food allergens have appeared in the literature. However, while OIT has been the most extensively studied form of food allergy immunotherapy, several drawbacks, such as the high adverse reaction rate (including anaphylaxis), disruption to lifestyle, and loss of protection after discontinuing therapy, have led to the search for more convenient and safer forms of therapy. In 1992, a brief report on the use of rush subcutaneous immunotherapy (SCIT) suggested the benefit of subcutaneous injections of peanut protein, but continued studies from the same group suggested unacceptable adverse side effects (3, 4). More recently, investigators have been evaluating sublingual immunotherapy (SLIT), epicutaneous immunotherapy (EPIT), and intralymphatic immunotherapy and biologics such as omalizumab and dupilumab, alone and in combination with OIT (5).

Among these emerging therapeutic options, EPIT is a promising approach that leverages the unique immune properties of the skin to induce a specific immunological response. Although the overall treatment goal of inducing desensitization is common to all forms of allergen immunotherapy (AIT), important differences exist in terms of efficacy, safety, and administration for each treatment modality. Each of these treatment characteristics is impacted by the route of allergen exposure and the underlying mechanism by which desensitization occurs. This review will examine in detail the mechanism, safety, and efficacy of EPIT as shown in clinical and preclinical studies, with a focus on its application in food allergy.

## Skin as a highly specialized immune organ

The skin is the largest organ of the body and is an incredibly complex, multifunctioning, and self-renewing organ playing multiple physiological roles. The most well-known function of the skin is to provide a physical barrier against mechanical damage, entry of foreign compounds, and ultraviolet light and prevent dehydration by retaining body fluids and moisture. However, the skin is not a simple inert and inflexible barrier. As a constant environmental sensor, the skin is a rather “selectively permeable” environmental interface able to constantly sense and dynamically respond to environmental stimuli.

### Skin structure overview

The ability of the skin to act as a multifunctional organ is closely related to its complex lamellar structure, which consists of an outer, non-vascularized epidermis overlying an inner dermis (6). Multiple strata of keratinocytes in the epidermis give the skin its resistance and rigidity, while the dermis mainly contains collagen fibers, which provide a structural framework and give the skin its elasticity and stretch. The human epidermis is composed of several layers of keratinocytes, at various differentiation stages, organized as four layers of proliferating cells, namely, the stratum basale, overlaid by several superficial layers of stratum spinosum and stratum granulosum (SG), which ultimately form the stratum corneum (SC), more commonly known as the “horny layer.” As the outermost layer of the skin, the SC is the first line of defense for the body and is largely responsible for the protective barrier that prevents unwanted materials from entering and excessive loss of water (7). The SC is composed of multiple layers of corneocytes, which are anucleate keratinocytes that have reached the final stage of keratinocyte differentiation. Corneocytes embed keratin filaments within a filaggrin matrix, and the cornified lipid envelope replaces the keratinocyte plasma membrane. Thus, the SC has a two-compartment structural organization in which corneocytes are embedded in a complex lipid matrix, prompting its comparison to a “brick-and-mortar” structure (8). Depending on the body location, corneocytes are stacked in up to 18–20 layers, and corneodesmosomes hold them together and are programed to go through a gradual degradation that enables the desquamation of the outermost layer of corneocytes. In the SG, cell–cell junctions called tight junctions (TJs) seal the keratinocytes together, forming a barrier that acts as a liquid–liquid interface and restricts the movement of molecules within the intercellular space. These TJs act as a “gate” for the passage of water, ions, and solutes through the paracellular pathway and serve to prevent excessive water loss and desiccation (9, 10). With the SC, TJs constitute the second physical barrier of the skin.

### Skin as an immune organ

Aside from acting as a physical barrier, the skin has also evolved into a highly competent immune organ densely populated by diverse and dynamic innate and adaptive immune effector cells that provide a robust first line of defense against exogenous threats, such as toxins and microbial pathogens (11, 12). Skin antigen-presenting cells (APCs), such as Langerhans cells (LCs) located in the epidermis and conventional dendritic cells (cDC1 and cDC2) in the dermis, are dedicated to the elicitation of an adaptive immune response through the capture and processing of antigens and the triggering of antigen-specific T-cell proliferation upon migration to draining lymph nodes and antigen presentation. The human epidermis may also contain CD8^+^ resident memory T (TRM) cells (a subset of non-circulating memory T lymphocytes that appear following the resolution of skin inflammation) and inflammatory dendritic epidermal cells (IDEC) (a subset of professional APCs that resides at the dermal–epidermal junction of lesional skin during skin inflammatory diseases) (13, 14). The dermis contains several other types of immune cells such as *γδ* T cells, innate lymphoid cells, CD4^+^ T cells, neutrophils, macrophages, and mast cells. This mixed population forms a complex and dynamic immune network allowing for the rapid induction of immune responses.

As the predominant cell population of the epidermis and being on the front line of defense, keratinocytes play a pivotal and strategic role in this system by acting as immune sentinels. Indeed, keratinocytes express a wide range of pathogen-associated molecular pattern (PAMP) receptors, such as toll-like receptors, allowing them to sense exogenous compounds and distinguish between dangerous pathogens and innocuous molecules or microorganisms (15). Keratinocytes can also sense and react to epidermal injuries by changing morphology and gene expression in response to mechanical insults, sometimes referred to as the “keratinocyte activation” state (16). Keratinocytes then initiate and orchestrate appropriate immune defense responses, such as the recruitment and activation of memory T cells and the activation/maturation of DCs, via the secretion of various proinflammatory cytokines, chemokines, and antimicrobial peptides, also referred to as “alarmins” (12, 17). This unique characteristic recently earned keratinocytes the nickname “cytokinocytes” (18).

Sir Edward Jenner established an interest in the skin as an interface between innate and adaptive immunities several centuries ago. Jenner's (19) work provided the very first scientific evidence that the skin can be used to generate an adaptive immune response to a pathogen—in this case, cowpox virus administered on scarified skin. Since this pioneering work, several groups, including ours, have confirmed that skin is a viable route for vaccination against pathogens and cancer and a relevant alternative to intramuscular or subcutaneous injections, provided that the skin barrier is altered by abrasion, microperforation, or tape stripping (20–29). Several comparative non-clinical and clinical studies reported that transcutaneous immunization can be as efficient or even superior to parenteral routes at inducing or reactivating specific T-cell responses (30–33).

In line with this unique ability to regulate specific immune responses to topical antigens, the skin likely plays pivotal roles in both allergen sensitization (34) and desensitization (35), depending upon the local inflammatory milieu, the state of the skin barrier, genetic predisposition, and timing of allergen exposure. It is not contradictory, but rather context-dependent, that the skin is capable of orchestrating these two very different immune responses. In terms of the capacity of the skin to promote allergen sensitization, the “dual allergen” hypothesis proposes that sensitization to food allergens may occur when the allergen is first presented to the immune system via an inflamed, eczematous skin, rather than orally, and arises from the observation that allergic individuals often react to their very first oral food allergen exposure, suggesting that prior sensitization had occurred. This hypothesis suggests that sensitization may occur in genetically susceptible individuals [including those with filaggrin mutations (36)] when the disrupted and/or inflamed skin (e.g., atopic eczema) is exposed to low-dose allergen prior to oral exposure, whereas early consumption/exposure to the same allergen leads to oral tolerance (37). Clinical studies have supported this concept showing that allergen-specific T cells express the skin-homing marker cutaneous lymphocyte antigen (CLA) in milk- and peanut-allergic individuals (38, 39). Although this hypothesis conceptually implies that restoring skin barrier functions by enhancing the level of skin hydration should prevent allergen sensitization, this has not been a universal finding (40, 41). In contrast to the immune response of the skin when presented with allergens in the context of inflammation and prior to other routes of exposure, there is now a large amount of evidence that applying allergen on intact and healthy skin areas can progressively lead to immune tolerance, rather than sensitization. The evidence for the capacity of the skin to orchestrate desensitization, in the context of sensitized individuals, and promote tolerance to allergens presented via the epicutaneous route will be examined in detail in the following sections of this review.

### Skin as a preferential route for tolerance induction

**Figure 1:**
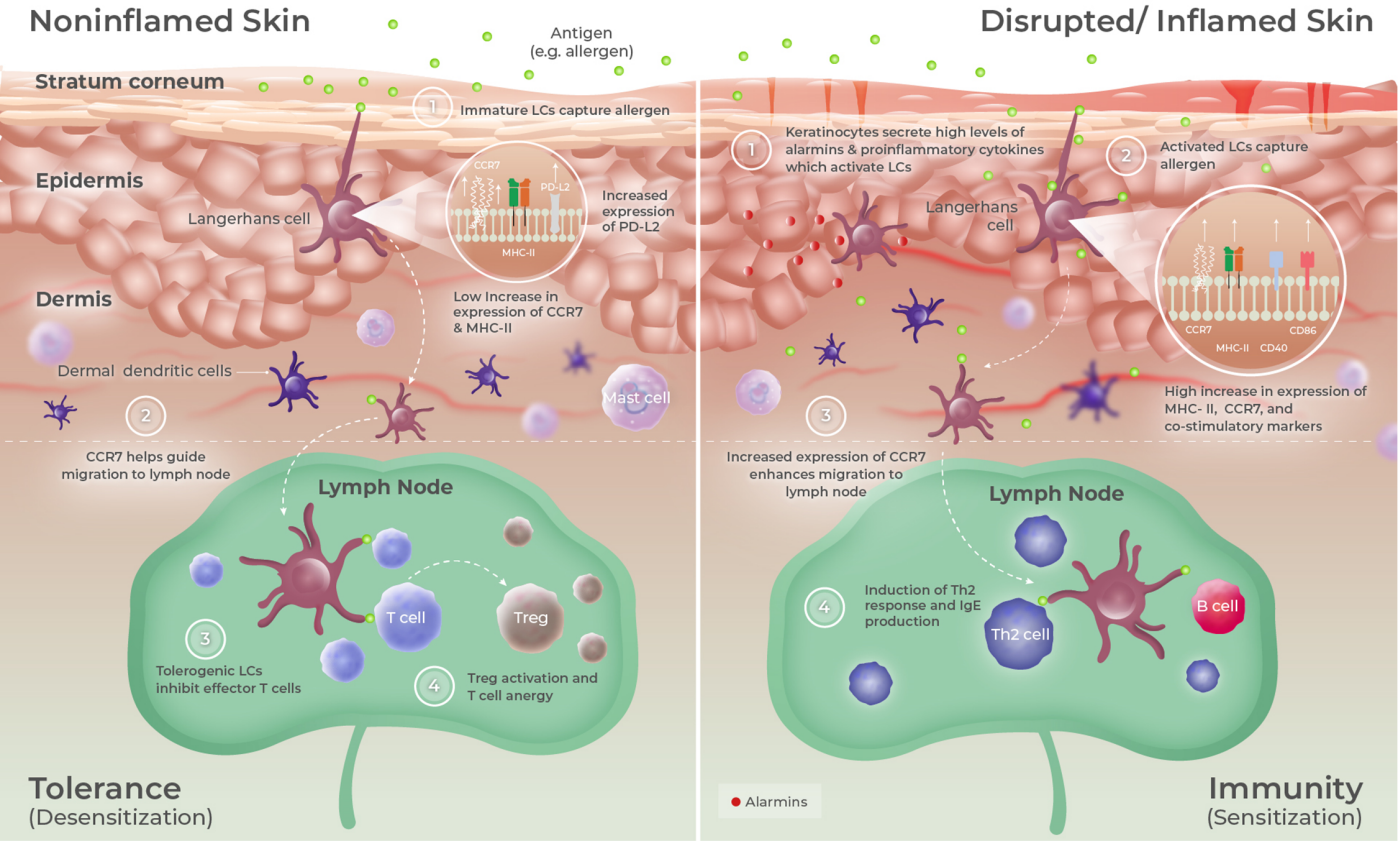
Epidermal microenvironment predisposes LCs to promote either tolerance or immunity. The skin is composed of three main layers: (i) the SC, composed of multiple layers of corneocytes corresponding to keratinocytes at a final stage of differentiation; (ii) the epidermis, a non-vascularized and rigid layer composed of multiple strata of keratinocytes; and (iii) the dermis that mainly contains collagen fibers. LCs are localized within the epidermis, where they can extend their dendrites to sample antigens having entered the stratum corneum. When taking up antigens under homeostatic conditions (left panel), LCs undergo a tolerogenic activation process characterized by the upregulation of MHC-II, CCR7, RelB, IL-12-p40, and PD-L2 expressions. Upon migration to skin-draining lymph nodes, those tolerogenic LCs preferentially induce Tregs or can promote T-cell anergy. Following injury, infection, or under inflammatory conditions (right panel), keratinocytes secrete high levels of alarmins and other proinflammatory cytokines that drive LC to acquire an immunogenic phenotype, mainly characterized by a strong upregulation of MHC-II and costimulatory molecules (CD40 and CD86) expression. Upon antigen capture and migration to skin-draining lymph nodes, those activated LCs preferentially induce effector T cells such as Th2 cells.

Surprisingly, the ability of the skin to promote specific immune tolerance has received minimal attention until recently. However, using the skin as a pro-tolerogenic route is a fairly old concept that was established in 1921, when Vallery-Radot and Hangenau (42) revealed that repeated applications of allergens onto the scarified skin reduced systemic allergic symptoms in patients allergic to horses. Only decades later, Senti et al. (43) and Dupont et al. (44) truly exploited this method of repeated antigen application to the skin (now referred to as EPIT) for the treatment of respiratory and food allergies, respectively. In fact, one pivotal role of the skin (and arguably the most important) is to maintain peripheral tolerance and prevent the elicitation of unwarranted immune reactions to harmless environmental agents, such as commensal skin bacteria or even self-antigens (45). Maintenance of tissue homeostasis and immune balance is indeed possible when antigens are sampled by immature and/or semi-mature DCs in the absence or low levels of keratinocyte activation and additional danger signals (17, 46). For instance, uptake of self-antigen by resident DCs leads to a tolerogenic activation process characterized by a slight increase in the expression of major histocompatibility complex (MHC) and costimulatory molecules (including CD80 and CD86), lymph node homing receptor CCR7, tolerogenic transcription factor RelB, and IL-12p40 cytokine, generating semi-mature migratory DCs (47). These “tolerogenic” DCs are unable to provide sufficient costimulatory signals to T cells, leading to T-cell anergy and/or the induction of specific regulatory T cells (Tregs) (Figure 1, left panel). In contrast, a stronger phenotypic maturation occurs when antigens are applied in an inflammatory context or concomitantly with the secretion of high levels of alarmins by keratinocytes, leading to the generation of DCs having acquired an “immunogenic” phenotype (Figure 1, right panel) (17). These activated DCs express high levels of MHC class II (MHC-II) and costimulatory molecules such as CD40 and CD86 and are thereby fully equipped to induce effector T cells rather than Tregs. Those elements perfectly illustrate the “Janus face” of the skin, which can efficiently induce a protective immune response in the case of danger (inflammation, skin barrier disruption, and pathogen invasion) or promote immune tolerance in other situations. Interestingly, there may be anatomical variations in cutaneous immunity. Del Duca et al. (48) illustrated this and analyzed immune cell infiltrates and gene expression profiles of the healthy skin collected from the interscapular upper back, the inner upper arm, the abdomen, and the outer upper thigh of 24 healthy individuals. The upper back contained the highest density of LCs, dermal DCs, and Tregs, suggesting that this skin location may have a particular ability to promote functional tolerance.

As the only professional APCs of the epidermis in homeostatic conditions and being at the forefront of sensing environmental components, LCs are key players in that immune process. LCs are a unique and fascinating population of immune cells derived from hematopoietic precursors sharing features with both macrophages and DCs (49). They are indeed formidable phagocytes that can self-maintain within the epidermis (like macrophages) but also migrate to skin-draining lymph nodes (sdLNs) to present processed antigens to T cells (like conventional DCs) (50, 51). LCs can elongate their dendrites to survey the external environment located just beneath the SC, from which they continuously capture and process self and foreign antigens (52). This unique feature has been remarkably illustrated in a mouse model by Kubo et al. (53), showing that LCs can “send” their dendrites toward the SC by reorganizing TJ structures, thus maintaining TJ barrier integrity during antigen capture. Like other skin DCs, LCs can promote either tolerogenic or immunogenic immune responses depending upon cytokine signals received from keratinocytes. For instance, murine and human LCs can be efficiently stimulated and activated by proinflammatory cytokines, such as tumor necrosis factor-α (TNF-α), IL-1β, or thymic stromal lymphopoietin (TSLP), leading to their maturation and switch into an immunogenic profile (54, 55). Of note, the release of these cytokines can be observed following skin barrier disruption in mouse models (e.g., using tape stripping) or in lesional skin areas during topical diseases such as atopic dermatitis, partially explaining why applying antigen on altered and/or inflamed skin can lead to efficient vaccination or sensitization in some cases (56–58). Conversely, data obtained from *in vivo* mouse models and *in vitro* clinical samples suggest that LCs migrating from the skin under quiescent conditions can promote a tolerogenic immune response mainly mediated by Tregs in local lymph nodes and skin, resulting in the suppression of allergic/inflammatory responses and restoration of homeostasis (59, 60). Kaplan et al. (61) initially demonstrated the crucial role of LCs in maintaining tolerance to exogenous compounds, showing that contact hypersensitivity to hapten was enhanced in transgenic mice lacking epidermal LCs. Since then, several lines of experimental evidence confirmed that LCs are mainly tasked to maintain peripheral tolerance under steady-state conditions and could even be pre-committed to that function (60, 62, 63). These data further reinforced the concept established by Vallery-Radot and Hangenau and revisited by Senti et al. (43) and Dupont et al. (44), whereby the “pro-tolerogenic” properties of the homeostatic skin, and more precisely the unique features of LCs at steady-state, can be advantageously leveraged to restore specific immune tolerance to allergens. This is precisely the purpose of EPIT as will be discussed in this review.

## Methods considered for epicutaneous allergen application

Since the skin constitutes a large and easily accessible organ, it has been widely used for drug administration. Transdermal delivery indeed appeared as a relevant substitute to oral administration since it bypasses gastrointestinal processing. Transdermal drug delivery systems (TDDS) have been designed in this respect, to promote the passage of small molecule drug substances through the epidermis and into the vascularized dermis to maintain an effective and sustained steady-state blood concentration following topical patch application (64).

However, it is important to understand that the current forms of EPIT being investigated are distinct from traditional transdermal drug delivery, whose efficacy is underpinned by specific pharmacokinetic and pharmacodynamic properties. Indeed, the aim of EPIT is not to use the skin as a transdermal delivery route to the systemic circulation, but rather to leverage the immune properties of the skin for induction of immune tolerance. In this regard, the concept of EPIT is aimed at promoting the passage of large proteins and/or allergens into the first layers of the epidermis, rich in pro-tolerogenic LCs. Targeting that highly immunocompetent area allows for the application of microgram amounts of allergen, whereas in comparison, OIT to food allergens uses dosage in the milligram/gram range. By targeting the epidermis, which is a non-vascularized tissue, EPIT also significantly reduces the risk of allergen passage into the bloodstream, thus limiting systemic allergen exposure and distribution. This aspect makes EPIT highly suitable for the treatment of potentially life-threatening allergies such as food allergies. Safety, tolerability, and treatment compliance are indeed among the primary treatment goals in EPIT since its ability to promote specific immune tolerance is based on repeated contact between the allergen and LCs over time, through regular application of allergens.

The first step of the immune pathway underlying EPIT involves the entry of the allergen into the SC and at the first living layers of the epidermis (SG), from which the allergen can be captured, internalized, and processed by LCs. However, this faces two interlinked challenges. First, the SC is theoretically impermeable to molecules having a molecular weight higher than 500 Da and should therefore constitute a major obstacle for the permeation of allergens that are mostly high molecular weight proteins (65). Second, any method aiming to promote the passage of allergen into the epidermis should prevent inducing excessive local inflammation to maintain LCs in a “quiescent” pro-tolerogenic state and limit the diffusion of the allergen toward the vascularized and mast cell-rich dermis to prevent the passage of free allergen into the bloodstream and minimize local reactogenicity. To achieve that goal, several strategies have been considered, including the use of coated microneedle patches, the application of allergen on skin pretreated by tape stripping, abrasion or laser-mediated microperforation, or the application of allergen on intact skin using an occlusive epicutaneous system (66–73).

Microperforation and tape stripping have been shown to greatly improve the passage of topical allergens to the epidermis compared to intact skin, potentially allowing for a reduction of the dose of allergen and a simplification of EPIT therapeutic schedules (i.e., reduction of the contact time with the allergen or the overall duration of the treatment). Such strategies could therefore constitute an attractive alternative to conventional SCIT for the treatment of respiratory allergies, as supported by efficacy data generated in human and/or animal models (67, 68, 72, 74, 75). Recently, Landers et al. (76) demonstrated in C3H/HeJ mice that the use of a topical stamp comprised of microneedles coated with low doses of peanut allergens was more efficient than the application of peanut allergen solution on intact skin to decrease allergic reactivity and intestinal mast cell infiltration following oral peanut challenge, suggesting that microperforation may also be adapted to the treatment of food allergies. This delivery platform is still in development under the acronym TASIS. In line with those results, Yu et al. (77) showed that the application of powder-laden, dissolvable, microneedle arrays containing a mixture of powdered peanut allergen, vitamin D3 (as a potent tolerogenic adjuvant) and CpG oligonucleotide (as a pro-Th1 adjuvant), can afford clinical protection against oral challenge in a mouse model of peanut allergy. However, it will be crucial to conduct further clinical investigations to truly assess the efficacy and safety of these technologies in humans.

In general, approaches relying on the disruption of the epidermal barrier may indeed risk facing important safety and tolerability concerns when addressing potent food allergens associated with a high risk of inducing anaphylaxis in allergic individuals. This aspect was well illustrated by Spina et al. (78) in an intraindividual controlled trial on 20 adults with birch pollen allergy, evaluating allergic skin reactivity induced by birch allergens applied epicutaneously following tape stripping or application of a microneedle array or delivered using a skin prick test (SPT). Both pretreatment with microneedles (100 µM length) and SPT led to immediate skin reactions in all subjects, suggesting that allergens rapidly diffused into the dermis where they promoted mast cell degranulation. In another study conducted on 98 grass pollen–allergic patients, von Moos et al. (79) compared the safety of EPIT performed either on skin prepared by abrasion or tape stripping. Systemic allergic reactions were experienced by six of 26 and one of 24 patients following abrasion or tape stripping, respectively. Beyond potential concerns about tolerability and adherence to treatment, local inflammation induced by epidermal barrier disruption could also interfere with immune tolerance induction, as suggested by data obtained from mouse models of allergy, where the application of allergen on intact skin promoted tolerance whereas similar application on tape-stripped skin further enhanced Th2 activation and sensitization (80, 81). In line with those results, data in mice showed that both tape stripping and microneedles can lead to efficient activation of skin APCs and notably LCs (82). Overall, additional clinical data are required to further evaluate the safety and the tolerability of “mechanical” skin barrier disruption for the epicutaneous application of potent allergens, since these parameters may have a significant impact on treatment compliance and/or outcomes.

Application of allergen on intact skin aims to optimize allergen delivery to the immune system while limiting systemic exposure to allergen. Occlusive approaches were developed for that purpose, to promote the entry of allergens within the superficial layers of epidermis without requiring skin barrier disruption. Indeed, as reported by Maibach and colleagues, the SC is known to be highly hygroscopic, which can significantly increase its water content, especially under occlusion (83–85). Notably, even a short-time occlusion of 30 min can result in significantly increased SC hydration (86). This excess of water can transiently disrupt the intercellular lipid organization leading to the dissociation of corneocytes and an increase in SC permeability. The SC could then potentially act as a reservoir ensuring a slow but sustained supply of allergens to LCs. This reservoir function of the SC has indeed been documented for a wide range of small molecules administered through conventional TDDS, allowing for a progressive release of the drugs for a sustained period following patch removal (87–90). In contrast to mechanical skin disruption, occlusion does not elicit skin irritation and has even been shown to decrease the expression of proinflammatory cytokines such as TNF-α and IL-1β in intact mouse skin, suggesting that this strategy is particularly adapted to the induction of tolerogenic LCs (91, 92). In that context, the University of Osaka developed an occlusive hydrophilic gel (HG) patch aiming to promote rapid and sustained hydration of the SC, thus enhancing the permeation of high molecular weight proteins (initially vaccine antigens) (93). This original epicutaneous device was recently investigated in an exploratory clinical pilot study aiming to evaluate the safety and efficacy of milk protein-containing HG patches in children with severe milk allergy (94). Among the eight enrolled children, only four experienced an increase in their cow's milk protein eliciting dose (ED) (the minimal amount of allergen that triggers an objective allergic reaction) following oral food challenge after 8–48 weeks of treatment, suggesting that further optimization is possible. Furthermore, placebo-controlled trials may help to fully assess the efficacy of this approach in milk-allergic children, for which the rate of spontaneous resolution is known to be high.

## Viaskin as the most clinically advanced approach to EPIT

**Figure 2:**
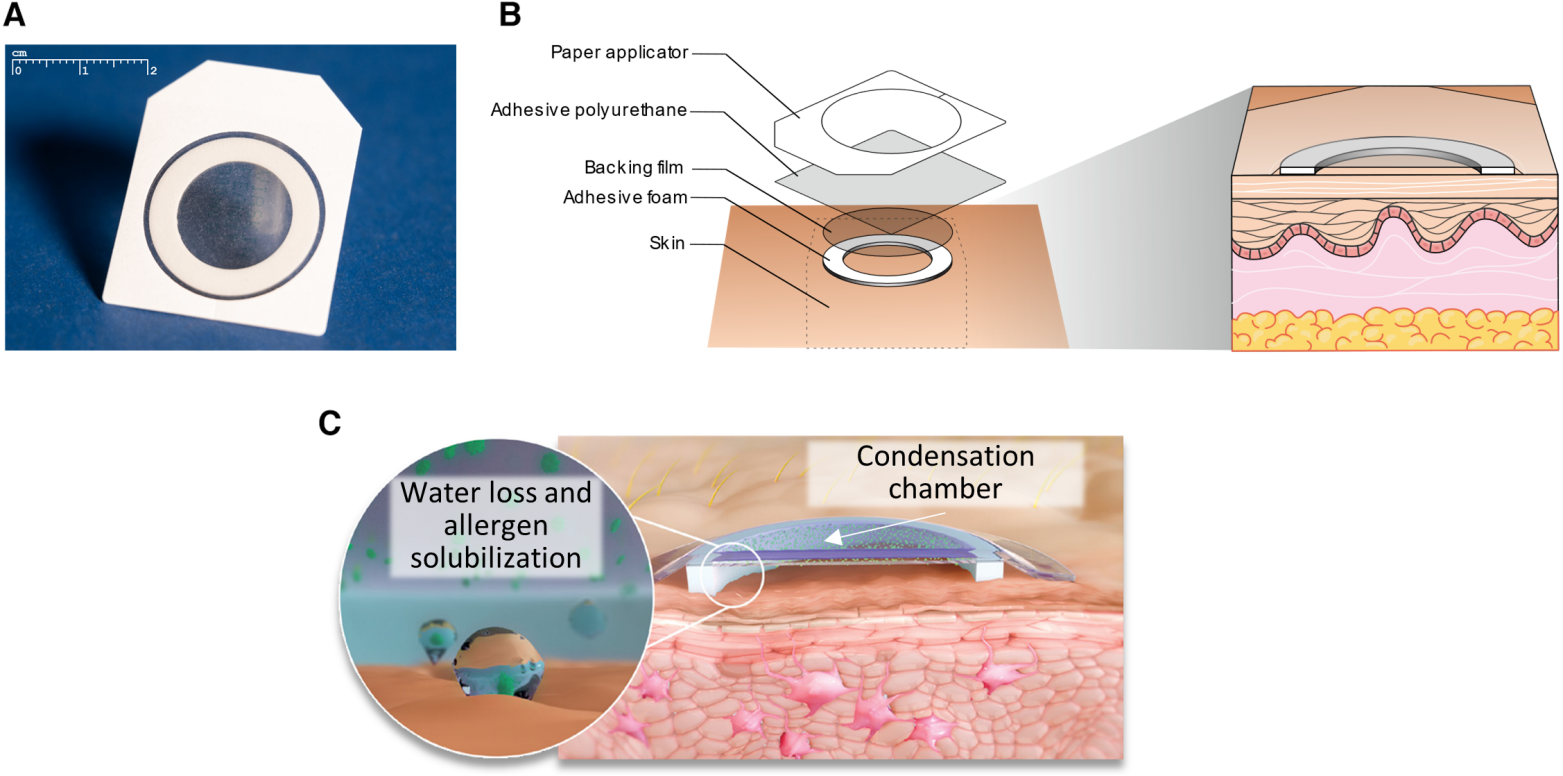
Viaskin epicutaneous system design and components. The Viaskin epicutaneous system (**A**) is an occlusive patch in which allergen is electrosprayed onto a backing film disc that is held above the skin with an adhesive foam ring and a flexible polyurethane adhesive dressing (**B**). This foam ring, when applied to intact skin, forms a condensation chamber that enables the natural water loss of the skin to solubilize and release the allergen from the backing film disc onto the skin to facilitate absorption into the epidermis (**C**).

**Figure 3:**
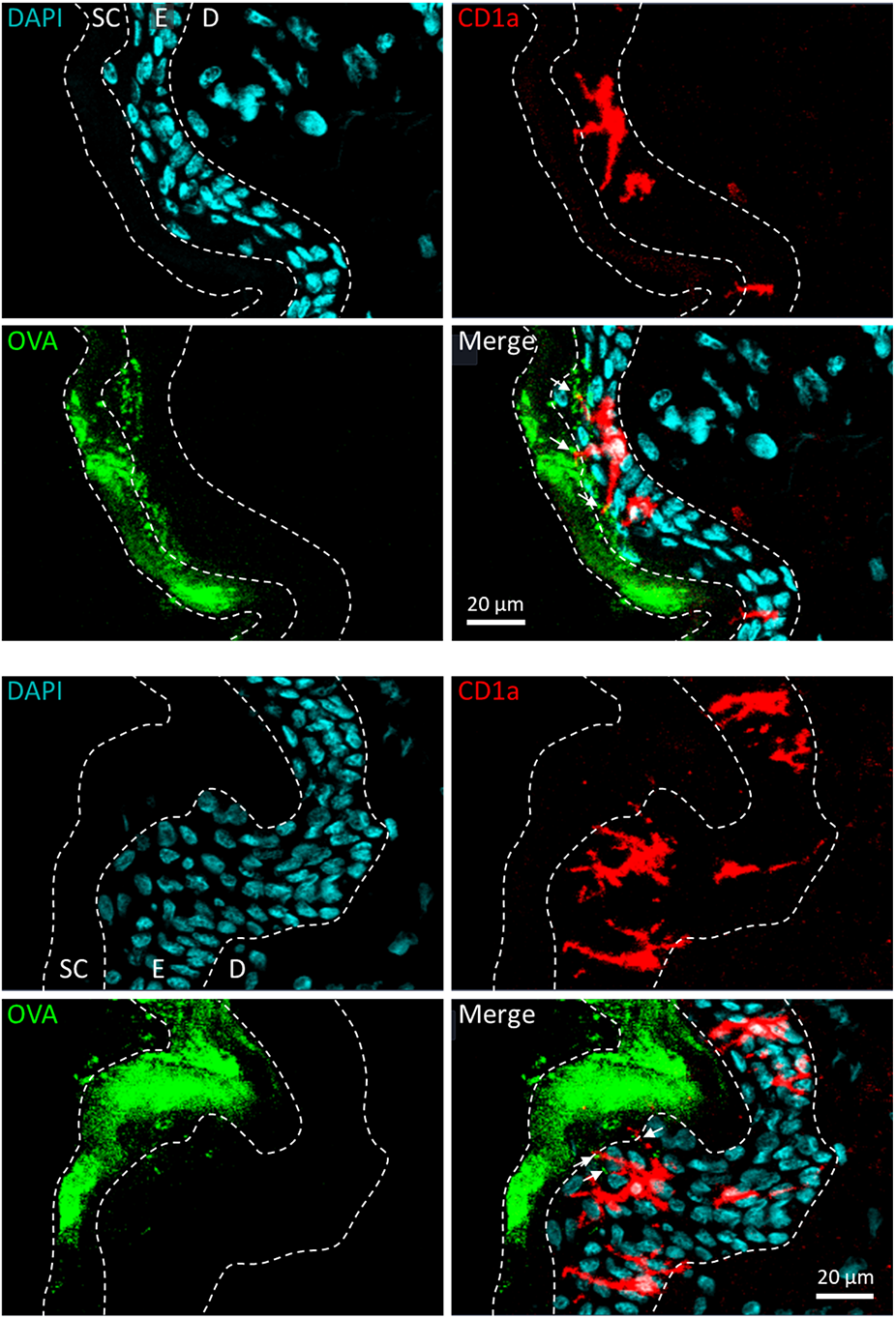
Allergen solubilization and capture following application of Viaskin to intact skin. Allergen applied epicutaneously under occlusion is mainly localized in the stratum corneum and the first layers of live epidermis, from which it is taken up by Langerhans cells through the extension of their dendrites. Here, Viaskin patches containing ovalbumin conjugated to Alexa Fluor AF488 (OVA, in green) were applied on live human skin explants *ex vivo* for 6 h. Frozen sections of skin were prepared and incubated with an anti-CD1a antibody associated with a relevant secondary antibody conjugated to Alexa Fluor™ 568 (CD1a, in red) and DAPI (in blue). Skin sections were visualized using a confocal fluorescence microscope at ×10 magnification. E, epidermis; D, dermis.

The most clinically advanced EPIT approach to date is the Viaskin technology platform. Viaskin is an occlusive epicutaneous system (patch) containing dried native allergen extracts, without adjuvants, which is applied onto intact skin (Figure 2A) (44). In this technology, the bioavailability of allergen is facilitated by the unique design of the system, in which allergen extract is electrosprayed onto a backing film disc that is held above the skin by a foam ring (Figure 2B). Together, the foam ring and the allergen-containing backing film disc form a condensation chamber, which enables the natural water loss of the skin to solubilize and release allergen proteins from the backing film disc onto the skin to facilitate absorption into the superficial layers of the epidermis (Figure 2C). A small fraction of allergen is then sampled by LCs through the extension of their dendrites (Figure 3). Before being studied in humans, the potential of this technology to address allergic diseases was thoroughly investigated in several mouse models of food allergy and allergic asthma, using a modified version of Viaskin adapted to the murine model. This modified Viaskin consists of a smaller adhesive and occlusive patch containing the dried allergen, applied on intact depilated skin (referred to as epicutaneous occlusive patch throughout this review). In a first proof-of-principle preclinical study, Mondoulet et al. (95) demonstrated that repeated applications of epicutaneous occlusive patches containing different allergens [including the food allergens ovalbumin (OVA) and peanut] were as efficient as SCIT for the prevention of allergic airway reactions in a sensitized murine model following aerosol allergen challenge. Following sensitization to OVA or peanut protein extract (PPE), mice were treated once weekly (100 µg total protein of each allergen) for 8 weeks with either one 48 h application of an epicutaneous occlusive patch containing the relevant allergen on intact skin (EPIT) or with one subcutaneous injection of allergen (SCIT) and compared with untreated mice (naïve) and mice treated with empty patches (sham) as negative controls. Mice sensitized to either OVA or PPE and treated with EPIT had a significantly reduced response to food allergen-induced bronchial hyperresponsiveness at levels similar to those seen in the SCIT and negative control groups. EPIT-treated mice also had decreased eosinophilic infiltration into the lung that correlated with decreased Th2 cytokines (IL-4, IL-5, and IL-13) in the bronchoalveolar lavage fluid (BAL) and serum and a modified IgG1/IgG2a ratio (the mouse equivalent of human IgG4 and IgG1, respectively). In a follow-up confirmatory preclinical study conducted in a mouse model of peanut allergy, EPIT with epicutaneous occlusive patches also reversed airway hyperreactivity measured by the invasive method of resistance/compliance following challenge with PPE, again with similar efficacy as SCIT (96). Furthermore, EPIT or SCIT induced a comparable and significant reduction in markers of allergic inflammation (i.e., eosinophils, eotaxin, and Th2 cytokines) in the BAL compared to the sham group, as well as a decreased IgG1/IgG2a ratio.

Tordesillas et al. (97) explored whether the route of primary allergen exposure was a factor in the outcome of immunotherapy in mice sensitized to OVA through the epicutaneous or oral route, followed by 8 weeks of EPIT (using epicutaneous occlusive patches) or OIT. At the end of the treatment, C3H/HeJ mice were orally challenged with OVA, and anaphylaxis was measured (evidenced by a drop in body temperature). Mice sensitized through the epicutaneous route and treated with either EPIT or OIT were completely protected from anaphylaxis. When mice were challenged again to evaluate sustained protection after 4 weeks without treatment, those treated with EPIT were still significantly protected against anaphylaxis whereas mice treated with OIT regained clinical reactivity. Furthermore, in mice orally sensitized to OVA, those treated with EPIT were completely protected from anaphylaxis, but those treated with OIT were not. While there was a significant increase in OVA-specific IgG1 and IgG2a levels after 8 weeks of EPIT, which persisted in skin-sensitized mice at week 12, functional assays did not support a role for antibodies in clinical protection, suggesting that the protection against anaphylaxis in this model was likely mediated by a mechanism other than blocking antibodies.

More recently, the capacity of EPIT to protect against cashew allergy was assessed in a murine model (98). Here, cashew-sensitized mice were treated for up to 16 weeks with epicutaneous occlusive patches containing cashew protein extract (one 48 h application on intact skin per week). EPIT was able to significantly increase the level of cashew-specific IgG2a throughout the therapy period. More importantly, treated mice were significantly protected against anaphylaxis, as demonstrated by the significant reduction of temperature drop and clinical symptoms observed following oral challenge. This was associated with a strong decrease in mast cell reactivity as shown by the reduction of mMCP-1/7 in plasma, suggesting that EPIT specifically decreased IgE-mediated anaphylaxis.

The clinical development program of Viaskin has mainly focused on the treatment of IgE-mediated peanut allergy, particularly in the pediatric population. EPIT treatment in this disease context aims to induce sufficient desensitization to an allergen, such that the risk of allergic reactions, and particularly severe allergic reactions upon accidental exposure/ingestion of the allergen, is significantly reduced. In this respect, the treatment aims of EPIT are not dissimilar to those of other forms of AIT (such as OIT or SLIT) or biological treatments targeted at inducing desensitization. However, the route of allergen exposure, adverse event (AE) profiles, modifications of treatment during activities of daily living such as exercise and intercurrent illness, and frequency of attendance at specialist clinics/rooms for monitoring vary widely between treatment modalities. For instance, compared to OIT, EPIT for peanut allergy (Viaskin Peanut) has fewer lifestyle restrictions due to treatment and a lower rate of withdrawals due to side effects (99).

Multiple clinical studies have been conducted aimed at assessing the efficacy and safety of Viaskin Peanut (referred to here as peanut patch), with more than 1,400 peanut-allergic trial participants exposed to the peanut patch across the development program to date (Table 1) (100–107). These include three phase 3 efficacy and safety studies in children, i.e., the EPITOPE study in peanut-allergic children aged 1–3 years, the PEPITES study in peanut-allergic children aged 4–11 years, and the REALISE study, a safety-only study in children aged 4–11 years that was designed to approximate real-world use. These studies followed promising data arising from four supportive phase 2 studies and four phase 1 studies. To reliably assess desensitization (increases in ED) in these randomized, double-blind, placebo-controlled studies, efficacy outcomes were based upon baseline and peri/posttreatment double-blind, placebo-controlled food challenges (DBPCFC), a method recognized as the gold standard for the diagnosis and assessment of food allergy and recommended by the US Food and Drug Administration (FDA) for use in food allergy treatment clinical trials.

**Table 1:** Summary of completed clinical trials with Viaskin peanut.

Study name (NCT)	Study design	Population	*N*	Treatment duration	Primary objective(s)	Primary reference
Phase 1						
PEP01.09 (NCT01170286)	Randomized, double-blind, placebo-controlled, dose-escalation	Peanut-allergic individuals aged 6–50 years	100	2 weeks	To assess the safety and tolerability of repeated applications of DBV712 in adult, adolescent, and child subjects allergic to peanut	N/A
SOLAR	Open-label	Healthy adults aged 18–25 years	30	24 h	To assess the amount of residual peanut proteins on the Viaskin Peanut epicutaneous system after application in adult healthy volunteers	(100)
BIOPOT (NCT03352726)	Open-label	Peanut-allergic individuals aged 12–50 years	20	24 h	To assess the biological potency of Viaskin Peanut-IHRP^a^ by skin prick test	N/A
EVOLVE	Randomized, double-blind, placebo-controlled	Peanut-allergic children aged 4–11 years	45	3 months	To assess the potential impact of modifications to the epicutaneous system and the IFU^b^ on the parent/caregiver and patient user experience	N/A
Phase 2						
VIPES (NCT01675882)	Randomized, double-blind, placebo-controlled, dose-finding	Peanut-allergic individuals aged 6–55 years	221	12 months	To assess the efficacy of three doses of Viaskin Peanut in peanut-allergic subjects	(101)
OLFUS-VIPES (NCT01955109)	Uncontrolled, open-label, follow-up study to VIPES	Subjects who completed VIPES	171	Up to 36 months	To assess the long-term safety and efficacy (based on DBPCFC^c^) of Viaskin Peanut in peanut-allergic subjects	(101)
CoFAR6^d^ (NCT01904604)	Randomized, double-blind, placebo-controlled, dose-finding	Peanut-allergic individuals aged 4–25 years	74	12 months	To assess the efficacy of two doses of Viaskin Peanut in peanut-allergic subjects	(102)
ARACHILD^d^ (NCT01197053)	Randomized, double-blind, placebo-controlled	Peanut-allergic children aged 5–17 years	54	6 months	To assess the efficacy and safety of Viaskin Peanut in peanut-allergic children and adolescents	(103)
Phase 3						
EPITOPE (NCT03211247)	Randomized, double-blind, placebo-controlled, dose-finding	Peanut-allergic toddlers aged 1–3 years	413	12 months	To assess the efficacy and safety of Viaskin Peanut in peanut-allergic toddlers after 12 months of treatment	(104)
PEPITES (NCT02636699)	Randomized, double-blind, placebo-controlled	Peanut-allergic individuals aged 4–11 years	356	12 months	To assess the efficacy and safety of Viaskin Peanut 250 µg in peanut-allergic children after 12 months of treatment	(105)
PEOPLE (NCT03013517)	Uncontrolled, open-label, follow-up to PEPITES	Subjects who completed PEPITES	298	Up to 36 months (with optional 2-year extension)	To assess the long-term efficacy (based on DBPCFC) and safety of DBV712 250 µg in peanut-allergic children	(106)
REALISE (NCT02916446)	Randomized, double-blind, placebo-controlled	Peanut-allergic individuals aged 4–11 years	393	6 months (followed by up to 36 months open-label)	To assess the safety of Viaskin Peanut 250 µg after 36 months of treatment in peanut-allergic children	(107)

^a^ IHRP, in-house reference preparation.

^b^ IFU, instructions for use.

^c^ DBPCFC, double-blind, placebo-controlled food challenge.

^d^ External study not sponsored by DBV Technologies.

Data from the phase 2b VIPES study showed that of the three doses tested (i.e., 50, 100, and 250 µg), the 250 µg dose displayed the greatest efficacy, with a greater effect noted in younger participants aged 6–11 years, relative to adolescents/adults (101). Those results are consistent with other studies on food allergy, suggesting possible greater plasticity of the allergic immune response earlier in life (108). Based on these findings, the 250 µg dose was selected for further clinical development and was used in the pivotal phase 3 studies, i.e., EPITOPE and PEPITES. During these trials, the primary measure of treatment effect was based on the percentage of treatment responders, defined as improvements in ED to peanut at DBPCFC between Month 0 and Month 12 from either ≤10 to ≥300 mg (approximately one peanut kernel) or from >10 to ≥1,000 mg (approximately 3–4 peanut kernels). In PEPITES, 35.3% of 4- to 11-year-old peanut-allergic children treated with the peanut patch were responders after 12 months of treatment, compared to 13.6% of children receiving placebo [*P *< 0.001, risk difference of 21.7% (95% CI, 12.4–29.8)] (105, 109). An even greater treatment effect was seen in the 1- to 3-year-old group in EPITOPE, with a responder rate of 67% in the peanut patch group vs. 33.5% in placebo [*P *< 0.001, risk difference of 33.5% (95% CI, 22.4–44.5)] (104, 110).

In addition to increases in ED, clinical trial data demonstrated reductions in the severity of allergic reactions occurring during DBPCFC, an important treatment goal for many peanut-allergic patients (104, 105, 111). Reaction severity during DBPCFC was assessed based on prespecified symptoms, the severity of which was graded by study investigators, and included objective symptoms in skin and upper and lower respiratory, gastrointestinal, and cardiovascular/neurologic systems. After 12 months of treatment, nearly twice as many participants treated with the peanut patch had a maximum symptom severity of “none” or “mild” compared with placebo participants (31.1% vs. 16.5%, respectively), and fewer subjects treated with the peanut patch had a maximum severity score of “severe” across these systems (16.2% vs. 27.5% of placebo participants, *p *= 0.019). There is also evidence from a small group of patients in both VIPES and PEPITES that EPIT with Viaskin may induce sustained unresponsiveness in a subgroup of patients (106, 112). In both studies, a subgroup of eligible participants treated with the peanut patch for 36 months underwent 2 months without treatment, followed by a DBPCFC. In each study, a similar proportion of children maintained desensitization to at least 1,440 mg of peanut protein after 2 months without treatment, 73.7% (14 of 19) of children aged 6–11 years in VIPES and 77.8% (14 of 18) of children aged 4–11 years in PEPITES.

Evidence of immunomodulation has been observed across all clinical studies to date, with increasing allergen-specific IgG4 during the first 12–18 months of treatment, which then plateau and stabilize at above baseline values, along with initial small elevations in allergen-specific IgE that peak at Month 3 of treatment and then fall to below baseline by 12 months of treatment (101, 104–107).

Throughout the development of the Viaskin Peanut patch, a consistent safety profile has been demonstrated across all age groups. Pooled data from 630 children aged 4–11 years enrolled in two phase 3 studies (PEPITES and REALISE) treated with the peanut patch for up to 36 months demonstrated the most frequently reported treatment-emergent adverse events (TEAEs) were mild to moderate local application site reactions that decreased with frequency and severity over time (106, 107). Serious TEAEs considered related to treatment were experienced by 0.8% of peanut patch participants and no placebo participants; 3% and 0.5% of peanut patch and placebo participants, respectively, experienced TEAEs leading to permanent study discontinuation. In children treated with the peanut patch for up to 36 months, a low occurrence of treatment-related anaphylactic reactions was observed (3.8%).

Consistent with the phase 3 studies in children aged 4–11 years, the most commonly reported treatment-related TEAEs in children aged 1–3 years (EPITOPE) were mild or moderate application site reactions (104). The rates of treatment-related serious TEAEs were very low (0.4% in the peanut patch group and 0% in the placebo group). Similarly, low rates of treatment-related anaphylaxis (peanut patch, 1.6%; placebo, 0%) and treatment-related epinephrine use (peanut patch, 1.2%; placebo, 0%) were observed. The tolerability of the peanut patch in toddlers is evidenced by low discontinuations due to AEs (3.3%) and high compliance rates that were comparable between treatment groups (97.0%). Taken together, these data provide evidence that EPIT with Viaskin Peanut, if approved, may provide a convenient, non-invasive method for safely desensitizing children with peanut allergy.

The clinical efficacy and safety of other forms of AIT targeted at the treatment of food allergy have recently been well described (110, 113–115). Direct comparisons between treatment modalities are difficult due to differences in study methodology, including the definition of clinical endpoints, and no head-to-head studies have been performed comparing EPIT with other forms of AIT, such as OIT or SLIT.

## Proposed mechanism of action of EPIT

As previously discussed, the skin contains many different populations of immune cells that can potentially interact with topically applied allergens during EPIT. Among those cells, professional APCs have been demonstrated to be the main players. These APCs can capture allergens through a variety of mechanisms, such as “non-specific” uptake by constitutive macropinocytosis and “specific” uptake via receptor-mediated endocytosis and phagocytosis (116). Of note, this internalization pathway may differ from one allergen to another as different APC surface receptors could be implicated in the capture. For instance, Langerin (a C-type lectin receptor expressed at a high level by LCs) has been shown to bind to OVA and participate in its uptake (81). Interestingly, data obtained from mouse models also suggest that the capture of topical allergens by APCs and their migration toward sdLNs could be significantly impacted by the preexisting immunological status of animals. Indeed, the capture of OVA by skin DCs following the application of an epicutaneous occlusive patch was more efficient and occurred more rapidly in OVA-sensitized mice than in naïve animals (117). This phenomenon was thought to be partly mediated by OVA-specific antibodies present in sensitized animals, since passive transfer of serum from sensitized mice increased capture of allergen and migration of APCs in naïve mice, suggesting that the Fc receptor on LCs could participate in allergen capture and processing (118). More studies are needed, however, to decipher the different receptors implicated in allergen capture by LCs.

Specific skin APCs are known to exert specialized functions (119). For instance, LCs are considered to be mainly specialized in tolerance induction while cDC1 is mainly specialized in cross-presentation and the induction of cytotoxic T lymphocytes (CTLs). Although the skin microenvironment can strongly influence the function of APCs, independently of their specialization, it is still crucial to understand which APCs are responsible for allergen uptake during the early events of EPIT. Increasing the depth of allergen penetration by epidermal barrier disruption (i.e., using laser microperforation or microneedles) could potentially have a strong impact on the type of APC populations that capture the allergen. However, only a handful of studies have been conducted to precisely address this question. In one of those studies, van der Burg et al. (120) used microneedles of different lengths (targeting either the epidermis or the dermis) to deliver OVA. They showed that deeper passage of allergen resulted in an increased migration of dermal DCs toward sdLNs but did not impact the migration of LCs. In another study, Yu et al. (77) showed that peanut allergens delivered using a dissolvable microneedle array are mainly captured by “immunoregulatory” macrophages expressing IL-10 and TGF-β. Note that in that case, potent tolerogenic adjuvants were used. When applied onto intact mouse skin using an epicutaneous occlusive patch, an allergen is captured not only by LCs but also by dermal DCs (117, 121, 122). In that case, capture by dermal DCs was likely to result from penetration of the allergen into hair follicles (121). To date, only a few studies have aimed to identify which APCs are responsible for allergen capture in humans. Histological analysis performed on *ex vivo* human skin explants suggested that LCs are the main subset involved in the uptake of allergen following the application of the Viaskin patch on intact skin (123). This discrepancy between mouse models and human skin could be explained by major differences in skin structures, with humans having a thicker epidermis with multiple layers of keratinocytes and a lower density of hair follicles. Nevertheless, the mouse model has been of major interest in deciphering some of the immune mechanisms underlying the EPIT approach.

Although several groups have focused on the potential of skin-derived APCs to induce systemic immune tolerance to topical allergens, the underlying mode of action was almost exclusively characterized in murine models using the mouse-adapted Viaskin epicutaneous occlusive patch applied on intact skin. In mice that received an epicutaneous occlusive patch containing OVA, OVA-positive APCs migrated to sdLN within 24–48 h where they modulated allergen-specific immune responses. Whereas both LCs and dermal DCs were able to capture allergen and subsequently migrate to the sdLN, they appeared to play different roles in desensitization and tolerance induction. LCs have been identified as crucial in the promotion of tolerance by EPIT as their absence impaired the efficacy of the treatment. This was demonstrated in Lang-DTR transgenic mice, in which the injection of diphtheria toxin led to a specific and complete depletion in LCs. In that model, EPIT with epicutaneous occlusive patches failed to promote the induction of Tregs and protect these mice from clinical symptoms following allergen exposure (122, 124). Interestingly, this crucial role of LCs was also supported by Luo et al. (81) using a mouse model of skin-induced tolerance, where the inhibition of subsequent sensitization was abolished in animals lacking LCs. Data obtained from mouse models revealed that LCs undergo a phenotype change during EPIT, characterized by the differential expression of some key costimulatory/inhibitory markers. Indeed, migratory LCs having captured topical allergens applied using an epicutaneous occlusive patch showed an increased expression of PD-L2 and a concomitant decrease in CD86 expression, suggestive of an enhancement of their pro-tolerogenic functions (121, 124, 125). Indeed, PD-L2 is a high-affinity ligand for the programmed cell death 1 (PD-1) immune checkpoint inhibitor expressed by activated T cells, and PD-L2-expressing DCs have been shown to suppress Th2 responses and play a role in tolerance induction in several mouse models of allergy (126). Another noteworthy observation is that LCs seem to further gain pro-tolerogenic capacities during EPIT. This was suggested by data obtained in OVA-sensitized mice treated with repeated applications of epicutaneous occlusive patches containing OVA, showing that migratory LCs progressively increase their level of PD-L2 expression and their ability to induce Tregs during the first 3 weeks of treatment, while progressively losing their capacity to stimulate effector T cells (122). Apart from PD-L2, a role of TGF-β has been suggested by the increased expression of *α*Vβ8 integrin in migratory LCs having captured topical allergen (124). These differences between allergen-positive and allergen-negative migratory LCs pointed out a particular role of antigen capture and processing in EPIT, but further investigations are needed to completely understand how this change in LC phenotype translates into systemic tolerance.

The role of dermal DCs is more complex to decipher. The depletion of cDC1s in XCR1-iDTR mice had no impact on the protection afforded by EPIT against challenge-induced symptoms, suggesting that they have no role in tolerance induction (122). On the other hand, Tordesillas et al. (121) showed that PD-L2^+^ dermal DCs having captured topical allergens through hair follicles can migrate toward sdLN to prime latency-associated peptide (LAP)^+^ Tregs. The capacity of dermal DCs to induce Tregs has also been shown in two different mouse models treated with epicutaneous occlusive patches (122, 124). However, while LCs increased their capacity to induce Tregs during EPIT, the same was not true for cDC2 (122). Thus, the contribution of cDC2s during EPIT is likely secondary to that of LCs in those models, since the desensitization effect is largely abrogated in mice devoid of LCs. However, reduced migration of cDC2s was observed in the absence of LCs, making it difficult to ascertain any role of this population (124). Moreover, cDC2 progressively switched from induction of Th2 toward Th1/Th17 responses during EPIT and may therefore participate in the desensitization through the attenuation of Th2 responses (122).

**Figure 4:**
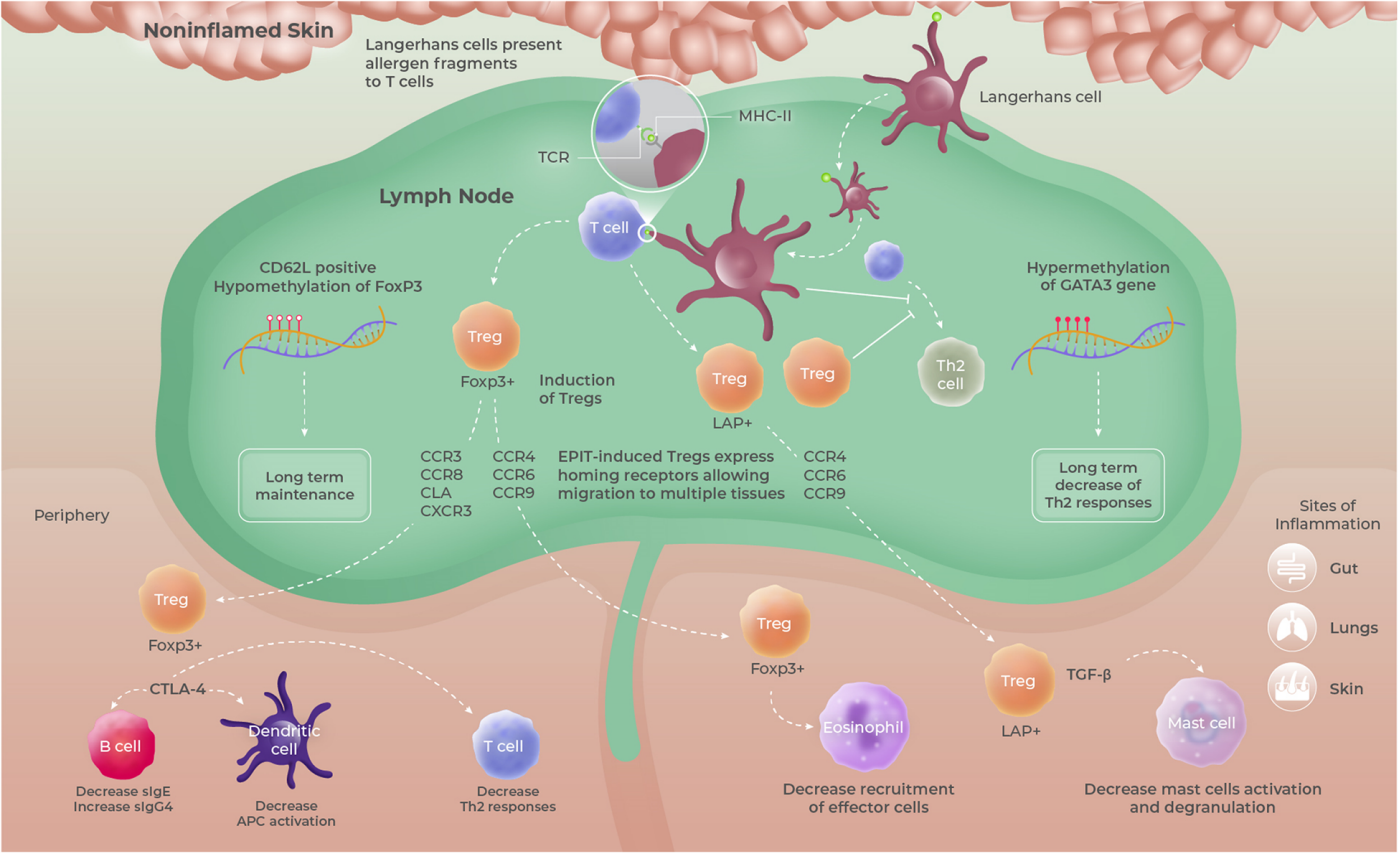
Proposed mode of action of EPIT, based upon data obtained in mouse models using epicutaneous occlusive patches. Allergen-positive Langerhans cells migrating from intact/non-inflamed skin promote the induction of long-lived FoxP3^+^ CD62l^+^ and/or LAP^+^ Tregs having the ability to migrate to multiple organs and tissues and sites of inflammation due to the expression of multiple homing receptors. Those Tregs then modulate allergen-specific immune responses through either cell–cell contact mediated by CTLA-4 or TGF-β secretion. Tregs, regulatory T cells.

Different immune mechanisms underpin the efficacy of AITs, involving various types of immune cells depending upon the modalities of AIT (i.e., schedule and route of administration, dose, nature of the allergen, and patient population) (127, 128). For instance, OIT is highly effective at promoting desensitization to food allergens through a rapid and early decrease in basophil and mast cell activation and the modulation of the IgG4 to IgE ratio, but sustained remission is rarely observed (129). Successful desensitization was generally accompanied by a marked reduction in specific Th2 immune responses, often resulting from an induction of Th1 cells. Although EPIT has also been shown to efficiently modulate Th2 immune responses, this approach uses separate pathways to achieve that purpose and also possesses its own immune specificities, with variations depending on whether the allergen is applied on intact or altered skin. For instance, Landers et al. (76) showed that the use of allergen-coated microneedles allows for faster desensitization than the application of allergen on intact skin in a mouse model of peanut sensitization, through a rapid decrease in Th2 and an increase in Th1 systemic responses. However, whether microneedles can promote a systemic Treg response was not apparent. In contrast, EPIT performed on intact skin using epicutaneous occlusive patches progressively decreased Th2 responses through the induction of Tregs (97, 117, 122, 130, 131) (Figure 4). As discussed earlier, these differences between altered and intact skin could be partly explained by the targeting of different subsets of skin DCs, with a higher proportion of dermal DCs being engaged by the microneedle approach, but also by differential levels of APC activation. Interestingly, this capacity to induce Tregs appears to be recovered when microneedles are coated with strong pro-tolerogenic adjuvants (77).

Tregs play an indispensable role in maintaining immune homeostasis and peripheral tolerance and are considered essential in promoting tolerance to allergens (132). Remarkably, Tregs were found to be at the cornerstone of the immune pathway triggered by the application of allergen on intact skin in mouse models of allergy (97, 131). Indeed, the depletion of Tregs completely abrogated the ability of EPIT to promote tolerance in peanut-sensitized mice treated with epicutaneous occlusive patches (131). Conversely, protection against oral allergen exposure could be transferred to sensitized mice by transferring Tregs from EPIT-treated animals. Further preclinical investigations revealed that topical allergen applied on intact skin using epicutaneous occlusive patches induced Foxp3^+^ Tregs that exert their suppressive activity in a cell-to-cell contact manner through CTLA-4 (131). However, no impact on the IL-10-producing Tr1 population was observed (130). In addition, in the study described earlier by Tordesillas and colleagues in OVA-sensitized C3H/HeJ mice, EPIT with epicutaneous occlusive patches promoted the selective expansion of a unique population of gut-homing LAP^+^ Tregs (Th3) that protected against anaphylaxis by direct suppression of gut mast cell activation, through the secretion of TGF-β (97, 130) (Figure 4). Those data are supported by the results obtained in cashew-sensitized mice, showing that EPIT strongly reduced mast cell degranulation following oral challenge (98). Interestingly, a head-to-head comparison of EPIT with epicutaneous occlusive patches vs. OIT and SLIT in a mouse model of peanut allergy revealed that these three approaches induce Tregs of different types and quality. In particular, Tregs induced by EPIT expressed a much broader repertoire of homing receptors than OIT and SLIT (130) (Figure 4). SLIT significantly enhanced the expression of CCR4 that has been implicated in the homing of Tregs to the lung but failed to induce the expression of gut-homing receptors. Although both EPIT- and OIT-induced Tregs expressed gut-homing receptors CCR6 and CCR9, EPIT-induced Tregs expressed significantly higher levels of CLA, a potent skin-homing receptor. Moreover, only EPIT-induced Tregs displayed increased expression of CCR8, a well-known Th2 homing receptor, and eotaxin receptor CCR3, known to promote the migration of T cells to sites of eosinophilic inflammation, as well as to the skin and lung. These results support the fact that EPIT-induced Tregs, in contrast to SLIT- and OIT-induced Tregs, possess a unique aptitude to traffic to numerous sites of allergen exposure and suggest that EPIT provides a multifaceted approach.

Another noteworthy difference resides in the maintenance of the induced Tregs. Indeed, in mice sensitized to peanut, OIT and SLIT mainly induced short-lasting CD62L^neg^ effector Tregs, while EPIT with epicutaneous occlusive patches promoted the differentiation of both CD62L^pos^ and CD62L^neg^ Tregs, that were able to maintain their suppressive activity for prolonged periods after treatment discontinuation (130) (Figure 4). This maintenance of Tregs induced by EPIT, in contrast to OIT, allowed for sustained protection against anaphylactic reaction upon allergen exposure for prolonged periods after treatment discontinuation (97). Long-term differences could be attributed to more profound modifications such as epigenetic modulation. Investigations in peanut-sensitized mice demonstrated that EPIT, with repeated applications of epicutaneous occlusive patches containing PPE, leads to epigenetic modifications in splenic and peripheral blood T cells, characterized by a unique DNA methylation profile compared to OIT (133). More specifically, EPIT promoted hypermethylation (silencing) of the *Gata3* promoter (transcription factor involved in the differentiation and function of inflammatory Th2 T cells) and hypomethylation (enhancing) of Treg-specific demethylated regions (TSDR) of *Foxp3* (involved in the development of Tregs and their suppressive functions). Interestingly, this epigenetic signature persisted up to 2 months after the end of treatment. Of note, a decrease in the methylation level of the *Foxp3* TSDR is a prerequisite for stable expression of Foxp3 in Tregs and the acquisition of their suppressive phenotype (134–136). Although OIT also promoted the demethylation of *Foxp3* TSDR in splenic T cells, it had no impact on the *Gata3* promoter. Moreover, this modification was not sustained after treatment was discontinued. Of note, such hypomethylation of *FOXP3* has been suggested to be predictive of sustained unresponsiveness in AIT-treated patients (137). In addition, high-throughput sequencing in helper T cells isolated from peanut-sensitized mice further treated by EPIT or by OIT revealed early changes in miRNA expression profiles during treatment (138). Although modifications of some miRNAs involved in T-cell differentiation and function were found to be common between both EPIT and OIT, most changes were specific to each approach. More specifically, some miRNAs known to be associated with asthma or allergic disease, were modulated solely by EPIT (139). This study provides further evidence for the molecular alterations underlying the mechanism of action of EPIT and might yield further clues as to its ability to promote sustained unresponsiveness.

Clinical trials conducted with EPIT have mainly focused on efficacy endpoints as well as on plasma biomarkers, such as changes in allergen-specific sIgE and sIgG4, and few clinical studies have looked at the modulation of the T-cell compartment. In the CoFAR6 study (NCT01904604), Berin et al. (140) aimed to evaluate the dynamics of the T-cell response in the blood of 49 peanut-allergic individuals during EPIT using the Viaskin patch. In the treated participants, a significant decrease in the number of peanut-responsive type 2 cells was observed by 26 weeks of treatment compared to baseline. This was associated with a significant increase in the frequency of peanut-responsive IL-10^+^ T cells at 1-year posttreatment. However, EPIT did not alter the frequency of peanut-responsive Foxp3^+^ Tregs in blood. Of note, this study was unable to provide information about the frequency of suppressive T cells in mucosal tissues (gut, esophagus, or skin), thus making any comparison difficult with the data obtained from mouse models.

Although further clinical investigations are needed to precisely decipher the immune mechanisms occurring in humans, there is an abundance of preclinical evidence that EPIT, when performed on intact/non-inflamed skin using occlusion, relies on a complex and unique immunological pathway starting from allergen capture by epidermal LCs toward the induction of specific and systemic immune tolerance mediated by Tregs. Experimental data generated in mouse models using epicutaneous occlusive patches strongly support the fact that EPIT, in contrast to SLIT and OIT, has the unique capacity to induce long-lasting populations of Tregs with broad tissue-homing capacities. These Tregs can further circulate systemically and modify the epigenetic signature of the T-cell compartment, thus leading to a progressive but long-term and systemic immune tolerance.

In comparison to other forms of AIT, clinical trials conducted with OIT suggest that greater increases in the threshold of reactivity to peanut allergen may be achieved (99). This is related to the route of allergen administration, with OIT allowing for the delivery of allergens to the same sites as those encountered during allergenic food consumption. Therefore, OIT works primarily through continuous stimulation of effector cells (i.e., basophils and mast cells), leading to effector cell hyporesponsiveness (exhaustion) and a rapid increase in threshold reactivity to the culprit allergen (referred to as clinical desensitization) (141, 142). However, this direct engagement of anaphylaxis-triggering cells substantially increases the risk of developing severe AEs, and the immediate benefits of threshold reactivity generally rapidly wane following the discontinuation of allergen consumption. The goal of EPIT is to provide a safer and more long-lasting approach, whose clinical benefits would rely mainly on the gradual induction of allergen-specific immune tolerance. Although both EPIT and OIT have demonstrated their ability to induce allergen-specific Tregs in preclinical models, experimental data suggest that only EPIT leads to sustained, long-lasting immune modifications representative of systemic tolerance induction.

## Beyond allergy

As highlighted in this review, the skin is a formidable and easily accessible immune organ with a unique ability to promote immune tolerance. Consistent with this, investigators have tried to leverage the immune properties of the skin to address immunological disorders other than allergies, such as inflammatory or autoimmune diseases.

For instance, EPIT has shown promising results in mouse models of inflammatory bowel diseases (IBD). In particular, Dunkin and colleagues reported that the application of epicutaneous occlusive patches containing OVA as a model antigen can abrogate intestinal inflammation and colitis in three murine models of IBD, through the induction of IL-10 and TGF-β-secreting Tregs (143, 144). Although mechanisms for protection were antigen-non-specific and thought to be associated with a bystander effect, those studies support the fact that EPIT can induce polyfunctional Tregs with strong suppressive functions. More recently, the same group demonstrated that an epicutaneous exposure to a chimeric peptide containing epitopes from CBir1, a dominant flagellin antigen with a potential role in Crohn's disease in humans, can alleviate colitis in a T-cell transfer colitis mouse model colonized with flagellin-positive bacteria, through the induction of gut-homing Tregs (145). The ability of EPIT to control gut inflammation was also nicely illustrated in a mouse model of eosinophilic gastrointestinal disorder, where animals were sensitized orally to peanuts and subsequently exposed to peanuts via a specific feeding regimen (146). This sustained oral exposure led to severe esophageal eosinophilia and intestinal villus sub-atrophy, characterized by a significant increase in eosinophils influx into esophageal mucosa and a reduction of the villus/crypt ratio. When these mice received epicutaneous occlusive patches containing PPE, they experienced a reduction in esophageal eosinophilia, a decreased expression of Th2 cytokines (IL-5 and IL-13) and eotaxin in tissue, and an improvement of intestinal villus sub-atrophy (146). A similar protective effect of EPIT was shown in peanut-sensitized piglets presenting with severe eosinophilic gastritis induced by prolonged exposure to peanuts in their daily diet (147). Indeed, piglets that received a daily application of the Viaskin peanut patch for 3 months showed a significant decrease in mucosal inflammation and gastric mucosal lesions and reduced gastric eosinophilia (at least a 10-fold reduction) to reach a level that was similar to non-sensitized animals. Interestingly, treated piglets also had decreased eosinophil counts in skin tissue following atopy patch testing, again highlighting the ability of EPIT to have an impact on multiple sites of allergen exposure. Those positive results were further supported by a pilot study conducted in pediatric patients with milk-induced eosinophilic esophagitis. Here, Spergel et al. (148) showed that 9 months of EPIT using Viaskin patches containing cow's milk allergens can reduce maximum eosinophil counts in the esophagus following the reintroduction of milk to the diet. Overall, these results provide interesting perspectives for future studies.

The potential of EPIT to treat systemic autoimmune diseases has also been demonstrated through several preclinical and clinical studies. In a mouse model of multiple sclerosis (MS), epicutaneous application of myelin peptides has been shown to ameliorate the development of experimental autoimmune encephalomyelitis (EAE), notably through the induction of antigen-specific suppressive T cells (149–152). Interestingly, this approach was successfully applied by Walczak et al. (153) in a placebo-controlled clinical trial, where MS patients received a mixture of three myelin peptides (1 or 10 mg), regularly applied epicutaneously using an adhesive skin patch throughout a 1-year treatment protocol. A significant reduction in brain lesions and annual relapse rate was observed in the treatment arm, compared to placebo, with the maximal effect of the treatment being reached at the dose of 1 mg. The potential of EPIT to address autoimmune disease has also been illustrated in mouse models of arthritis. Indeed, Strid et al. (154) showed that EPIT using type II collagen applied on stripped skin could inhibit the development and progression of arthritis and could ameliorate ongoing disease in an animal model of chronic collagen-induced arthritis (CIA). The authors proposed that the main mechanism for protection was a skewing of collagen-specific response from Th1 toward Th2, this immune orientation probably having been promoted by the use of tape stripping prior to the application of type II collagen. Although levels of CD4^+^CD25^+^ Tregs were not increased in this study, inhibition of the proliferation of IFN-*γ*-producing T cells suggested that some Tregs were induced. The ability of EPIT to limit the severity of arthritis has also been confirmed by Marcińska and colleagues using a mouse model of CIA (155, 156). These studies showed that applying a gauze patch soaked with a solution containing type II collagen on intact skin can induce suppressive T cells able to regulate the evolution of the disease and prevent synovial inflammation. In particular, the authors demonstrated that a specific population of suppressive CD4^+^CD8^+^ROR*γ*t^+^ T cells was involved in protection. Overall, these studies suggest that EPIT could impact the course of autoimmune diseases by promoting immune tolerance to autoantigens, notably through the induction of specific suppressive T cells.

Although several studies suggest that EPIT-induced Tregs can have a broad and non-specific anti-inflammatory effect in the organs to which they are localized through bystander mechanisms, the selection of suitable (auto)antigens to be used in humans remains a key challenge for many diseases.

## Concluding remarks

The skin is a remarkable organ providing a highly effective barrier and an easily accessible immune organ with a unique ability to promote immune tolerance. Numerous preclinical studies have demonstrated that EPIT can leverage these properties and induce potent and long-lasting T-regulatory cells with broad homing capabilities, which can exert their suppressive effects in multiple organs and ameliorate immune responses from different routes of allergen exposure, i.e., lung, skin, and gut. Several different strategies have been utilized to circumvent the SC barrier and deliver allergens to the epidermis. Mechanical skin disruption and microperforation allow for a rapid and efficient entry of antigens/allergens into the epidermis but may present safety concerns when attempting to desensitize patients with life-threatening allergies. On the other hand, occlusion on intact skin relies on the progressive passage of smaller amounts of allergens into the SC through repeated application of the allergen but is much simpler to develop and has demonstrated an excellent safety profile.

Clinical trials with EPIT using the Viaskin Peanut patch have demonstrated a balance between efficacy and safety for the treatment of life-threatening allergies in younger patients, at an age when allergic diseases start to occur. There is clinical evidence of continued efficacy of the peanut patch in children following 1 year of treatment, during which time the skin is exposed to a total amount of peanut that is less than 100 mg (approximately the equivalent of one-third of one peanut kernel), highlighting the potency of the skin as an effective route of desensitization. Moreover, this treatment approach is designed as a convenient, non-invasive therapy with no restrictions on daily activities.

The use of the skin as a means of inducing a specific immune response has, rightfully so, been given increasing attention in recent years, resulting in the ongoing development of multiple approaches to EPIT. Advances in this field, at a time of increasing prevalence of allergic and autoimmune disorders, have the potential to provide improved treatment options for patients.
